# Preliminary study of indocyanine green-guided laparoscopic lateral lymph node dissection for rectal cancer

**DOI:** 10.1371/journal.pone.0307077

**Published:** 2024-07-15

**Authors:** Chu-Ying Wu, Wen-Jin Zhong, Kai Ye

**Affiliations:** Department of Gastrointestinal Surgery, The Second Affiliated Hospital of Fujian Medical University, Quanzhou, Fujian Province, China; University of L’Aquila, ITALY

## Abstract

**Background:**

LLNM can occur in mid-low rectal cancer, but LLND in patients with rectal cancer presents certain challenges. Recent years have seen the progressive application of ICG fluorescence imaging technology in colorectal surgery. This study aimed to explore the effectiveness of ICG-guided laparoscopic LLND for rectal cancer.

**Methods:**

We applied ICG-guided laparoscopic lateral lymph node dissection in 11 patients diagnosed as rectal cancer with lateral lymph node metastasis.

**Results:**

All 11 patients in this group successfully completed ICG-guided laparoscopic LLND for rectal cancer with good lateral lymph node imaging.

**Conclusions:**

ICG-guided laparoscopic LLND for rectal cancer is safe and represents a feasible solution, thereby providing valuable guidance for intraoperative lymph node dissection.

## Introduction

Rectal cancer is currently the third most common malignancy worldwide, and its incidence continues to rise. Lymph node metastasis is a main pathway for rectal cancer metastasis and also an important cause of recurrence and death inpatients after radical surgery for rectal cancer. With the development of surgical techniques in recent years, total mesorectal excision (TME) has become the standard surgical procedure for mid-low rectal cancer. Conventional TME does not involve lateral lymph nodes outside the rectal mesentery. However, in cases of locally advanced rectal cancers with Lateral lymph node metastasis (LLNM), lateral lymph nodes remain potential sites for local recurrence. As an effective treatment method for removing lateral lymph nodes in rectal cancer, Laparoscopic lateral lymph node dissection (LLND) can considerably reduce the local recurrence rate after rectal cancer surgery, leading to good prognoses. However, the complex anatomical structure of the lateral rectum increases the difficulty of intraoperative localisation and dissection. Indocyanine green (ICG) is a clinically used near-infrared imaging agent that can be applied intraoperatively for lymph node tracing and is known for its good feasibility and safety. This work retrospectively analysed the clinical data of 11 patients with mid-low rectal cancer who underwent LLND with the use of ICG during surgery, exploring the applicability and value of ICG fluorescence imaging technology in laparoscopic LLND for rectal cancer.

## Methods

### Patients

**Fig 1:**
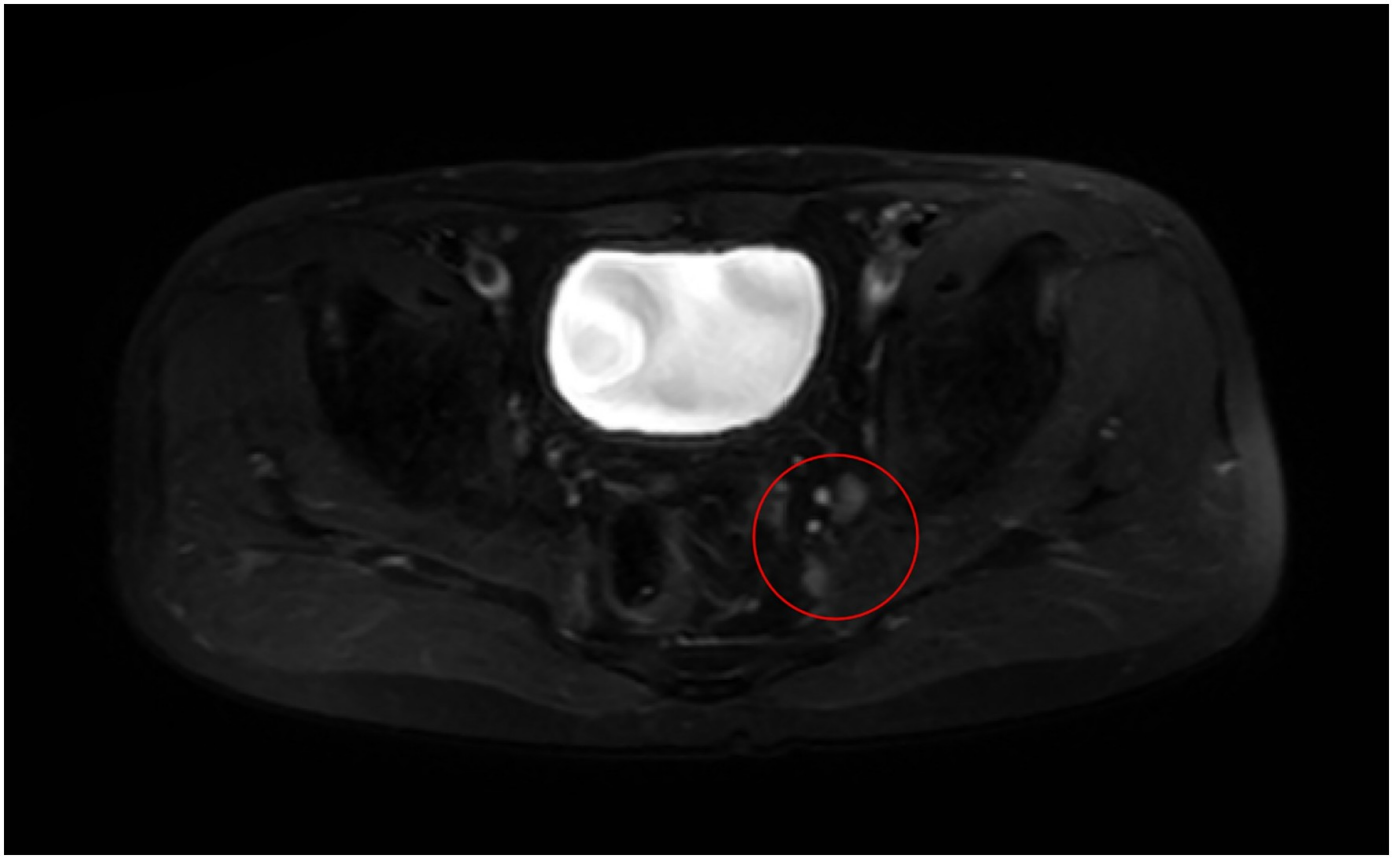
Preoperative MRI showed a short diameter of the lateral lymph node of the rectum of ≥10 mm.

This retrospective study evaluated the clinical data of 11 patients who underwent laparoscopic LLND for rectal cancer between 1 January 2022 and 30 June 2023 in our department. All data were fully anonymized. The data were accessed on 1 August 2023. The patients included 8 males and 3 females aged 44–74 years, with a median age of 61 years. The BMI of the patients ranged from 19.7 to 28.0 kg/m2, with a median of 20.9 kg/m2. The patients and their families signed informed consent forms for surgery, and the conduct of this study complied with the ethical requirements outlined in the Declaration of Helsinki when collecting patients’ clinical data. Indications for LLND in rectal cancer were preoperative colonoscopy and pathology showing rectal cancer (tumour located below the peritoneal reflection) and magnetic resonance imaging (MRI) showing a short diameter of the lateral lymph node of the rectum of ≥10 mm (Fig 1). LLND was not performed if 1. the patient had lateral metastatic lymph nodes invading the piriformis muscle, sacral plexus nerve or surrounding the iliac vessels; 2. LLNM was accompanied by distant metastasis that could not achieve R0 resection; or 3. highly extensive metastasis to lateral and retroperitoneal lymph nodes was present. All patients received ICG guidance during intraoperative lateral lymph node dissection.

### Ethics statement

**Fig 2:**
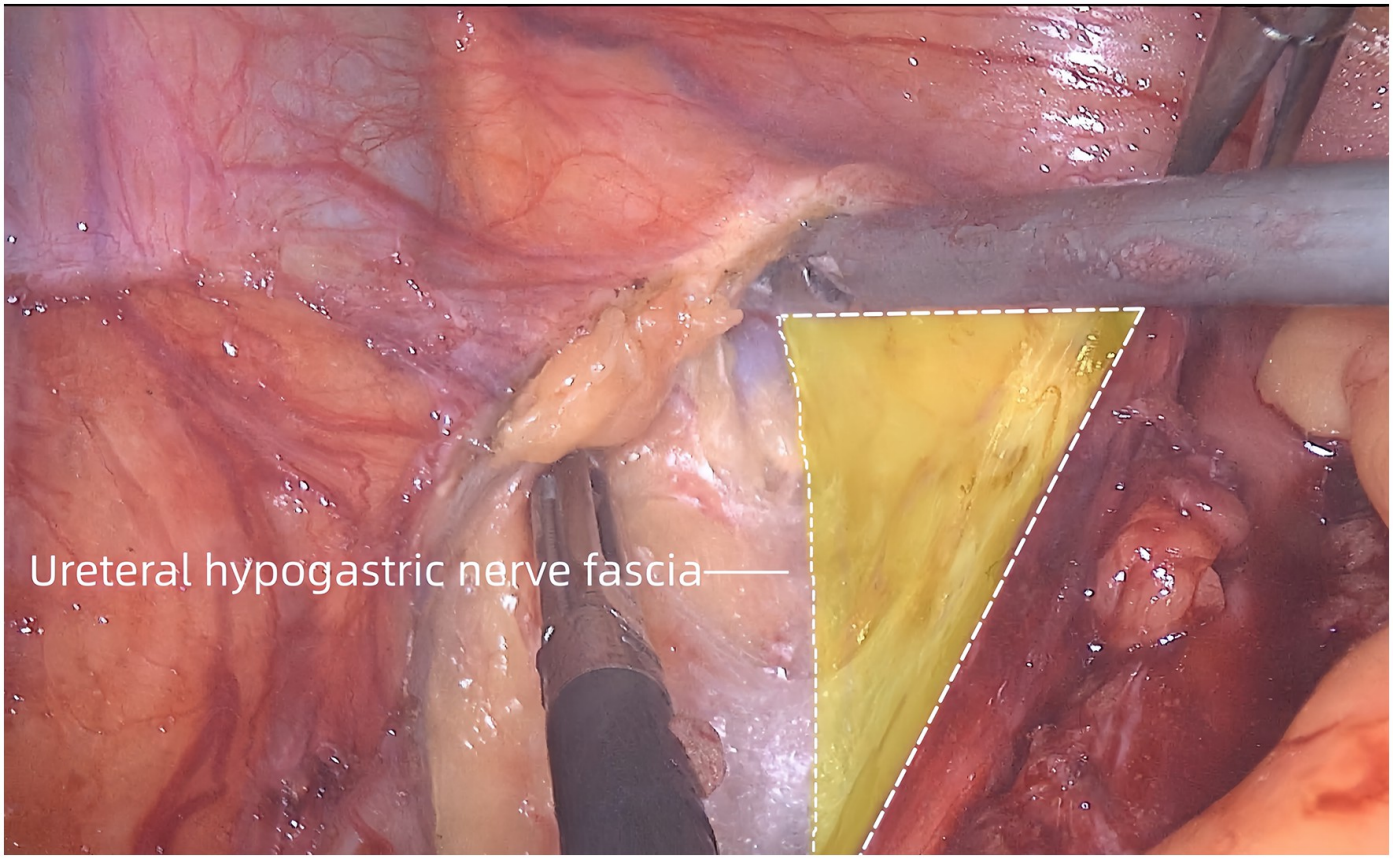
The ureteral hypogastric nerve fascia was exposed as the medial boundary for dissection.

**Fig 3:**
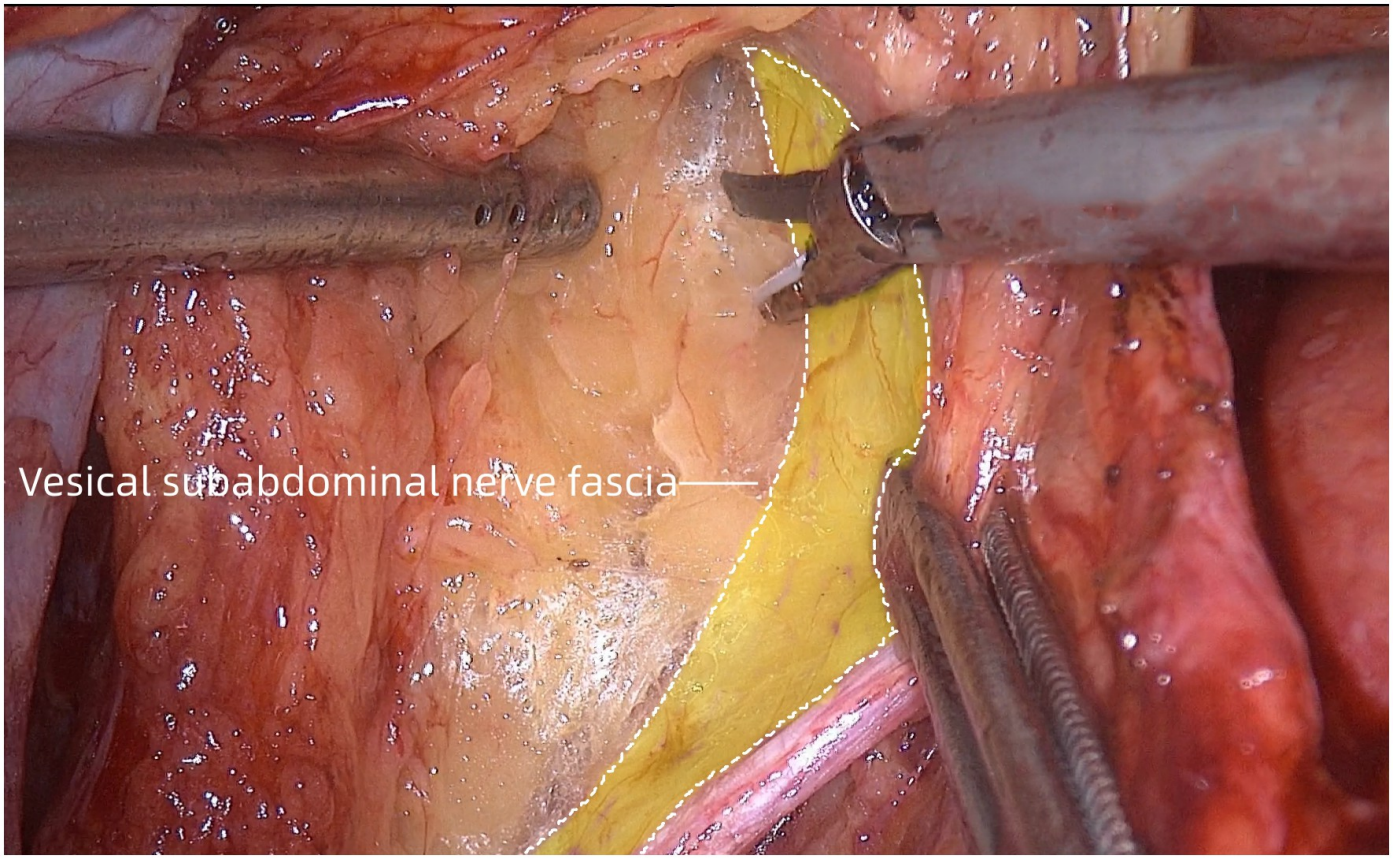
The vesical subabdominal nerve fascia was exposed as the lateral boundary for dissection.

**Fig 4:**
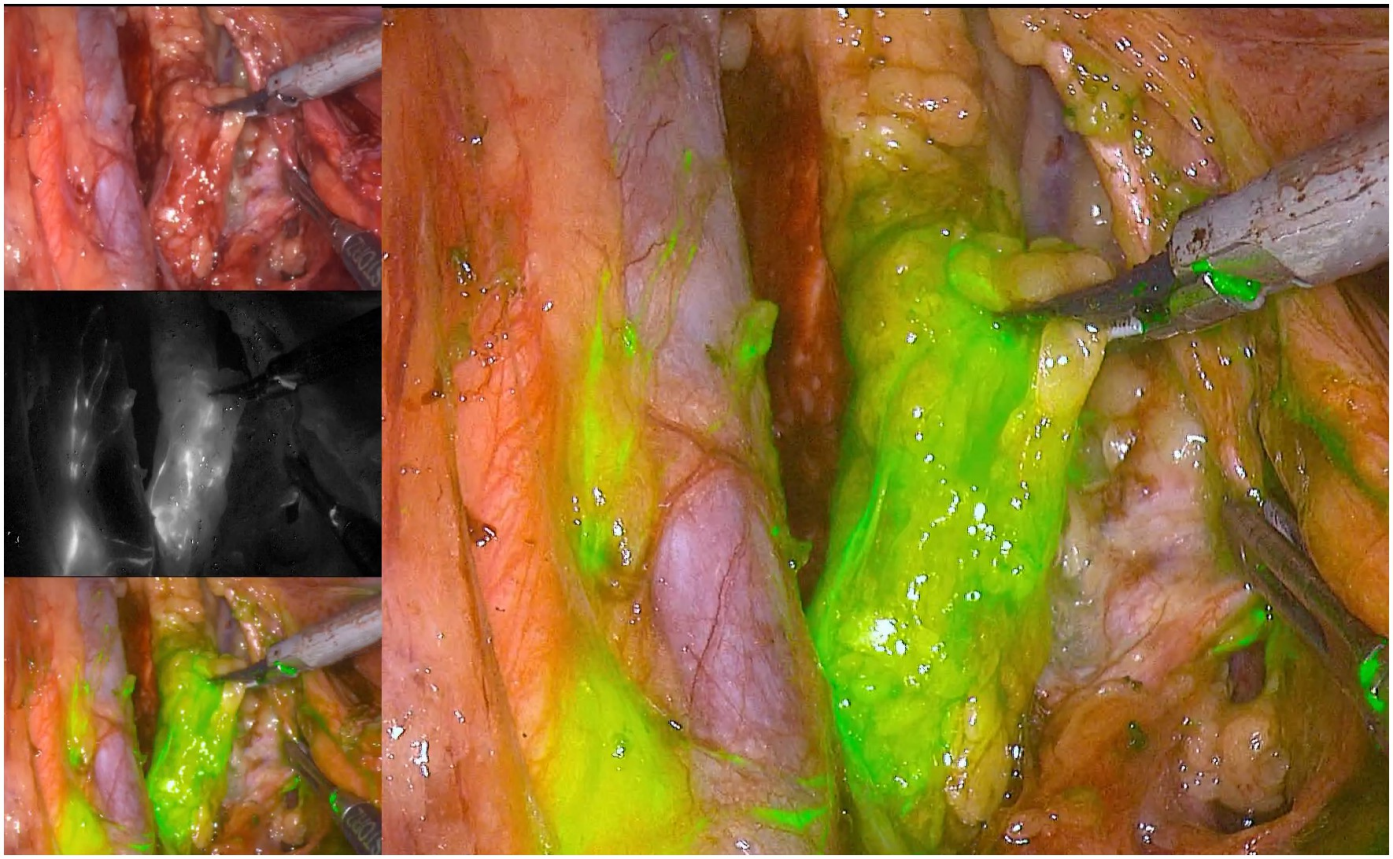
The obturator lymph nodes were located under ICG fluorescence imaging.

**Fig 5:**
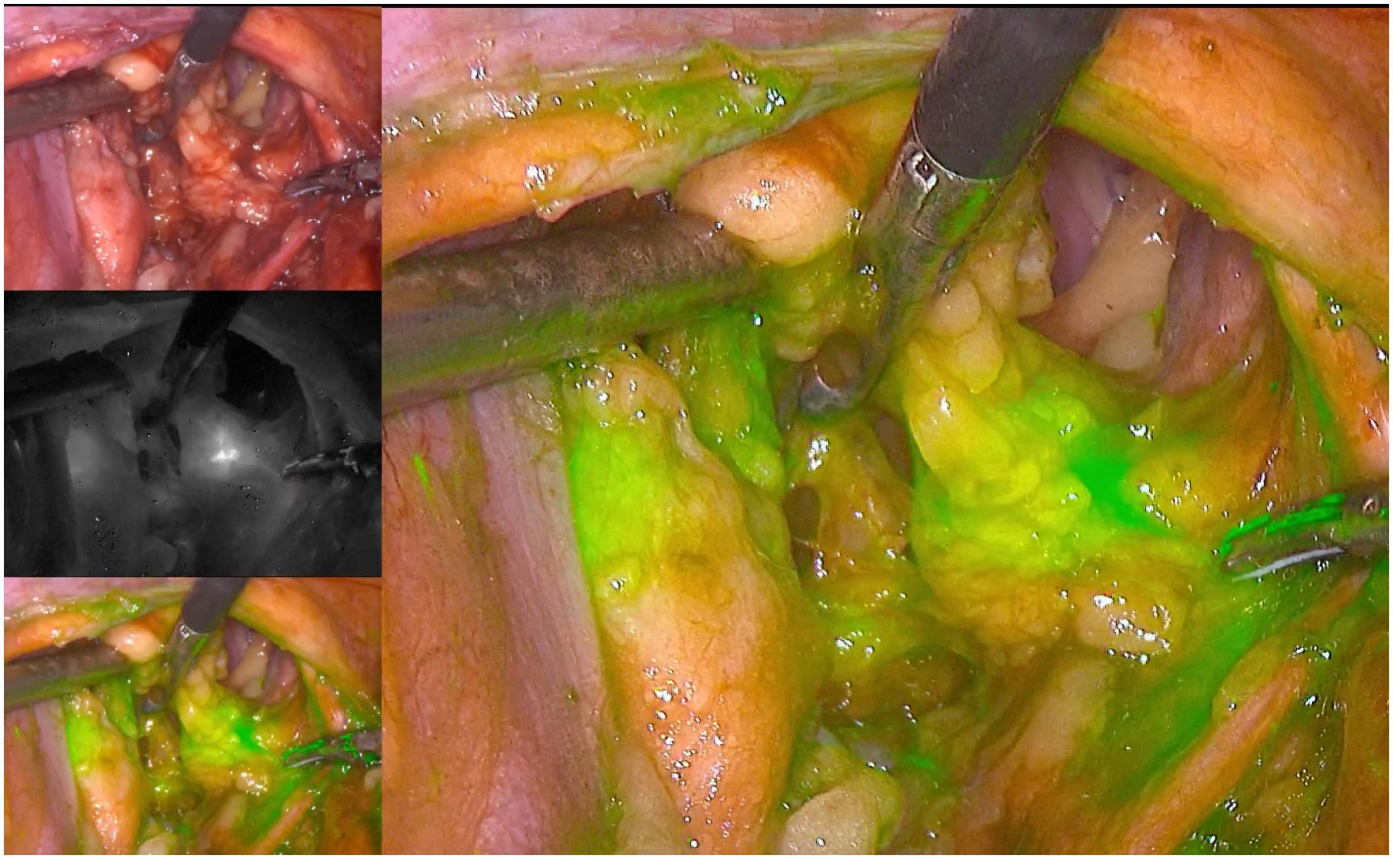
The internal iliac lymph nodes were located under ICG fluorescence imaging.

**Fig 6:**
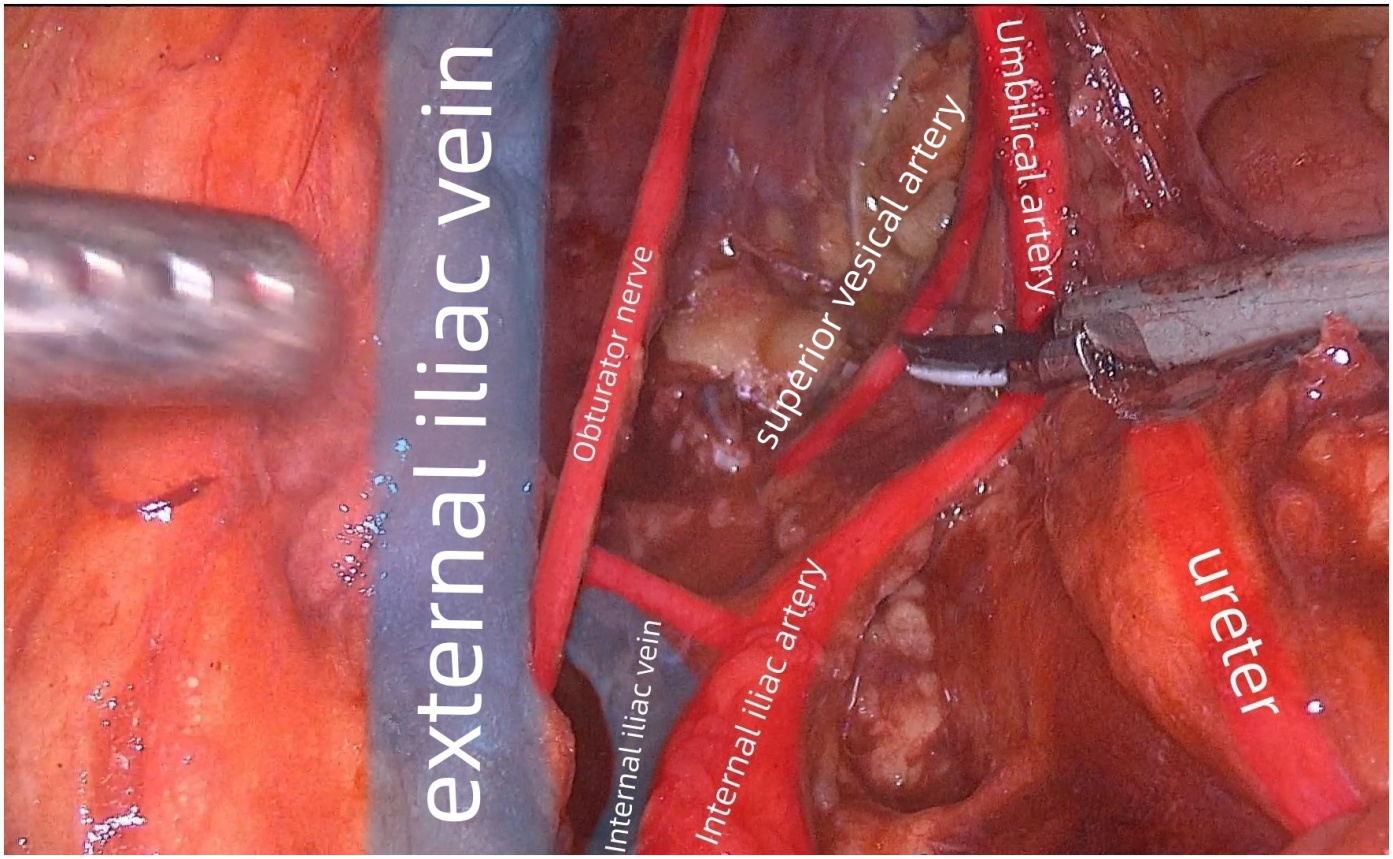
Surgical condition after LLND (under ICG fluorescence imaging).

**Fig 7:**
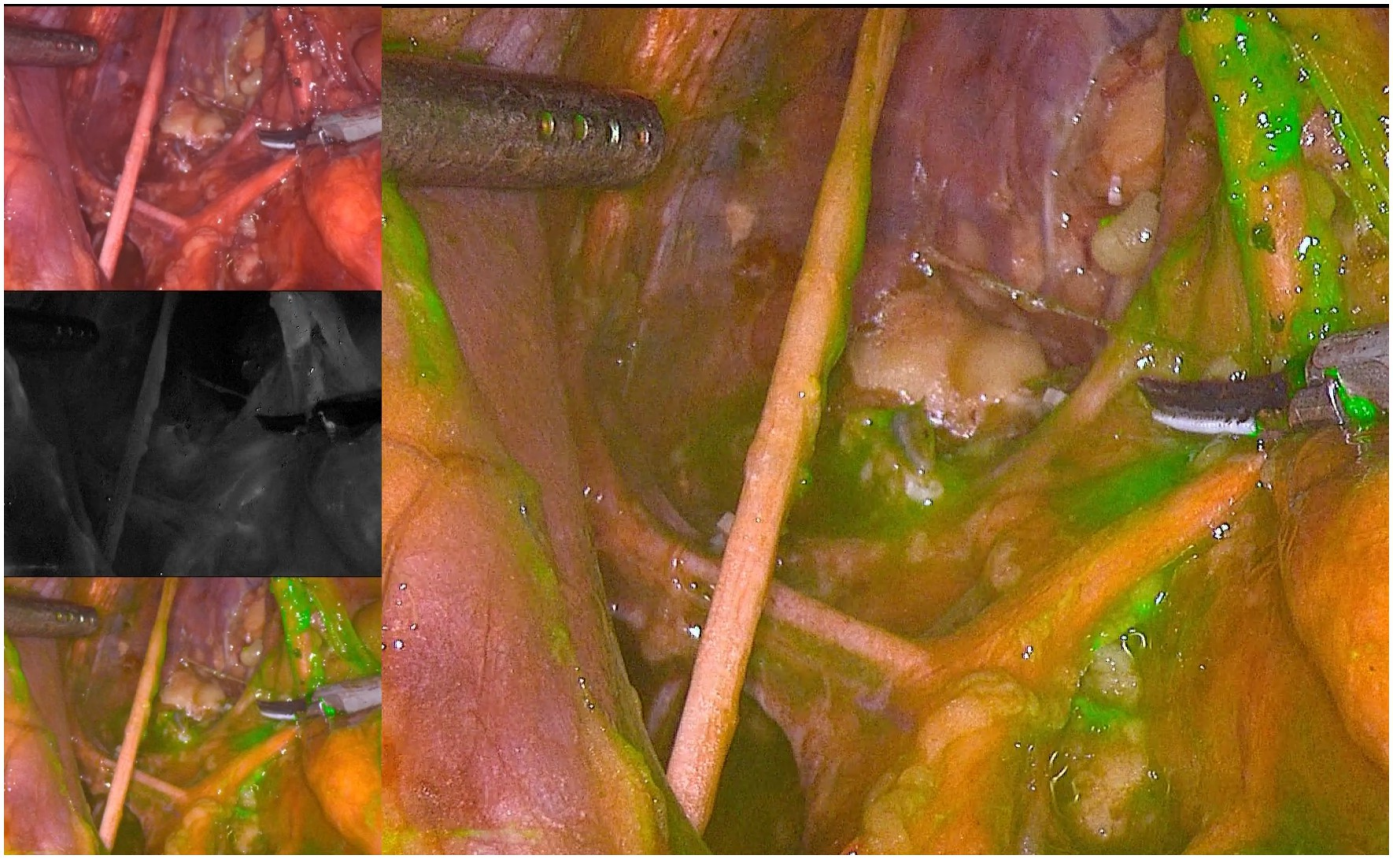
Surgical condition after LLND (under ICG fluorescence imaging).

**Fig 8:**
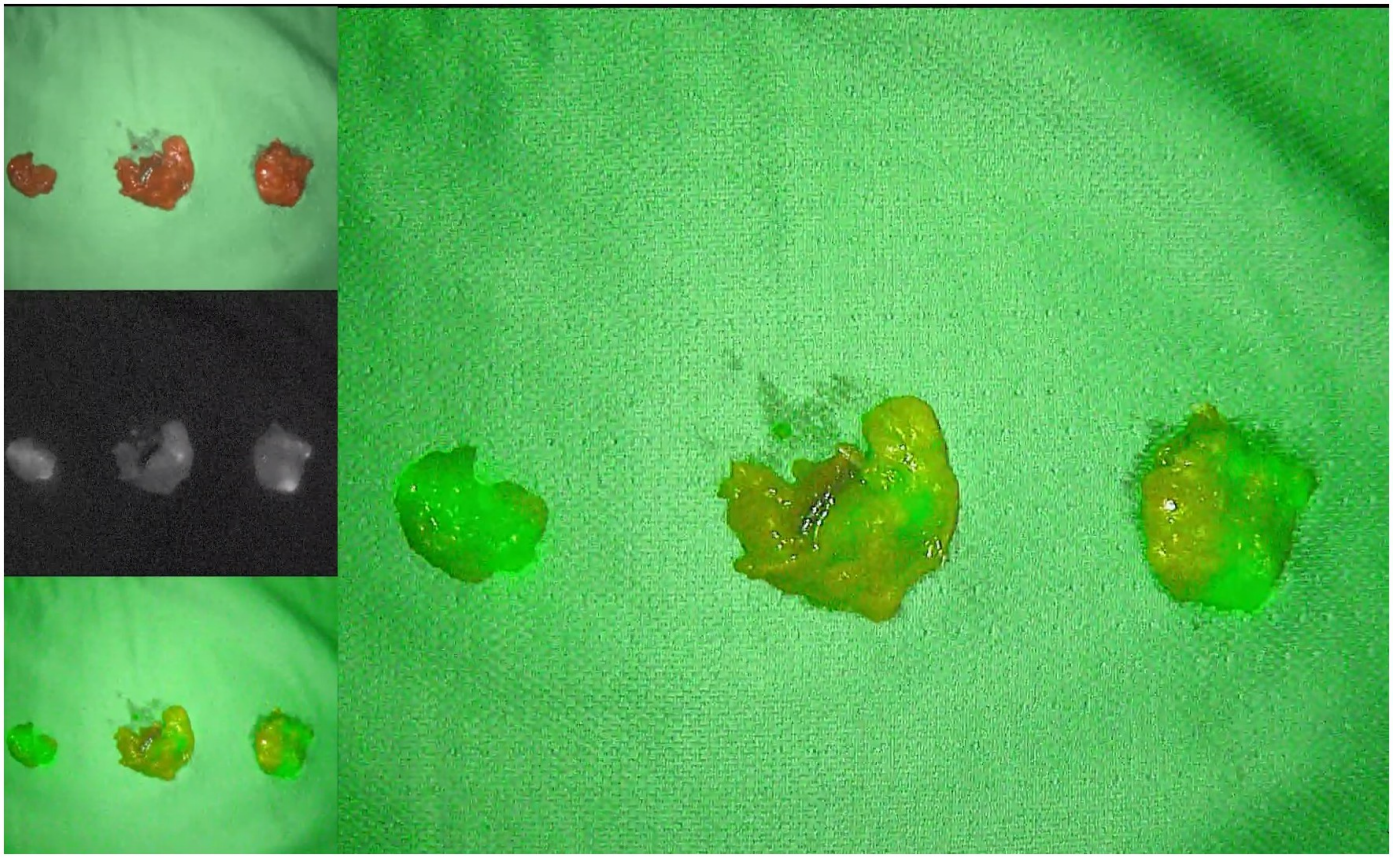
The postoperative ICG fluorescence imaging of the lateral lymph nodes, including the proximal lymph nodes of the internal iliac vessels (No. 263d), the proximal lymph nodes of the internal iliac vessels (No. 263p) and the obturator lymph nodes (No. 283).

This study was approved by the Ethics Committee of the Second Affiliated Hospital of Fujian Medical University. Because of its retrospective nature, the requirement of informed patient consent was waived. The individual pictured in Figs 1 to 8 has provided written informed consent (as outlined in PLOS consent form) to publish their image alongside the manuscript.

### Surgical procedure

In all patients, four injection points were established around the tumour under colonoscopy 12 h before surgery, with each injection point receiving a submucosal injection of 0.1 mL of ICG solution (2.5 g/L). A five-port laparoscopic approach was used for the surgery. The laparoscopic equipment utilised during surgery was a fluorescence laparoscopic system with a standard white light mode, real-time fluorescence mode and near-infrared light mode, all of which could be freely switched. The standard white light mode was a conventional laparoscopic high-definition imaging system, whereas the real-time fluorescence and near-infrared light modes could achieve intraoperative ICG fluorescence imaging and could be flexibly switched in accordance with the actual situation by the assistant holding the laparoscope. All patients underwent rectal TME surgery, with LLND being performed after the TME steps were completed and the rectum was transected.

The peritoneum was incised along the medial side of the internal iliacvein. After this procedure, the medial edge of the internal iliac artery and ureter was cautiously freed while taking care to protect the artery and ureter. The ureteral hypogastric nerve fascia was exposed as the medial boundary for dissection (Fig 2). A purse-string suture was used to pass below the ureter, the ureter was pulled towards the medial side, and the purse-string suture on the body surface was secured.

The sheath of the external iliac vein was opened along its medial edge, and the parietal peritoneum of the posterior peritoneum was deeply freed towards the medial side until entering the loose Toldt space on the surface of the iliopsoas and obturator internus muscles. The external iliac vein, iliopsoas and obturator internus muscles were revealed as the lateral boundary for dissection.

Dissection was performed along the umbilical artery. Then, the lymph nodes and adipose tissue around the umbilical artery were cleaned. The bladder, obturator lymph nodes and umbilical artery, as well as the vesical subabdominal fascia which also served as the lateral boundary for dissection, were revealed after sufficient freeing (Fig 3).

Dissection was continued along the umbilical artery towards the tail end. The main surgeon used intestinal forceps and an ultrasonic scalpel for blunt and sharp dissection to expose the obturator nerve while being careful to protect the obturator nerve. The obturator lymph nodes were located under ICG fluorescence imaging (Fig 4), the distal obturator lymph nodes were cleaned, and the obturator vein was transected.

Subsequently, the main surgeon returned to the proximal end of the internal iliac vessels. The starting point of the obturator artery was found, the obturator artery was clamped and transected after it was denuded, and the obturator lymph nodes and surrounding adipose tissue were cleaned while being careful to protect the obturator nerve and the internal and external iliac veins. Next, the main surgeon used their ultrasonic scalpel to continue to dissect downwards along the surface of the obturator nerve to clean the lymph nodes around the nerve. They then returned to the proximal end, continued to clean the surface of the sciatic nerve and transect the branches of the obturator vein and continued to clean the lymph nodes on the lateral side of the obturator nerve.

The internal iliac lymph nodes were located under ICG fluorescence imaging (Fig 5). The vascular sheath was opened along the course of the vein and dissected from the distal end to the proximal end between the internal iliac vein and ureter. The internal iliac lymph nodes were cleared, with special attention given to protecting the internal iliac vessels, pelvic nerve plexus and ureter. Then, dissection was continued along the internal iliac artery towards the distal end, exposing branches, such as the umbilical artery’s occluded part, superior and inferior vesical arteries and the distal branches of the internal pudendal artery. The superior and inferior vesical vessels were then dissected. After vascularising the above-mentioned vessels, the lymph nodes were cleared, the inferior vesical vein was ligated, and distal lymph node clearance was continued along the surface of the superior vesical artery. The cleared specimens were removed. Surgical condition after LLND was shown in Figs 6 and 7 (under ICG fluorescence imaging). The postoperative ICG fluorescence imaging of the lateral lymph nodes, including the proximal lymph nodes of the internal iliac vessels (No. 263d), the proximal lymph nodes of the internal iliac vessels (No. 263p) and the obturator lymph nodes (No. 283), is shown in Fig 8.

## Results

All 11 patients in this group successfully completed ICG-guided laparoscopic LLND for rectal cancer, with no case requiring conversion to open surgery. The total operation time was 153–198 min, the intraoperative blood loss was 14–27 mL, and the time for LLND was 28–44 min. Postoperative pathology showed that the number of lateral lymph nodes dissected was 3–13, and that of metastatic lateral lymph nodes was 1–4. Metastatic lymph nodes included eight metastases to No. 263d lymph nodes, six metastases to No. 263p lymph nodes and 10 metastases to No. 283 lymph nodes. All metastatic lymph nodes were identified by ICG during surgery except for one patients in which No. 263p lymph node was not identified. The reason why the lymph node did not show may be because of the obstruction of lymph node return due to the cancerous embolus of the lymphatic vessel. All patients were discharged 6–7 days postoperatively, and no complications occurred during surgery or within 30 days postoperatively. Clinicopathological data of all patients are presented in Table 1.

**Table 1:** Clinicopathological data.

	Sex	Age (y)	BMI (kg/m^2^)	Total operation time (min)	Intraoperative blood loss (ml)	Time for LLND (min)	Number of lateral lymph nodes dissected	ICG-identified lymph nodes			Metastatic lateral lymph nodes			Postoperative hospital stay	pT stage	pN stage
								No. 263d	No. 263p	No. 283	No. 263d	No. 263p	No. 283			
**Patient 1**	Male	61	20.3	169	15	30	7	1	1	0	1	1	0	7	3	1b
**Patient 2**	female	44	22.8	184	19	37	12	2	0	1	2	1	1	6	4a	2b
**Patient 3**	Male	69	20.9	183	18	36	13	1	2	1	1	2	1	7	4a	2b
**Patient 4**	Male	53	22.8	191	24	43	5	0	1	1	0	1	1	6	2	2a
**Patient 5**	Male	57	20.8	197	27	43	3	1	0	0	1	0	0	6	2	1a
**Patient 6**	Male	61	28	167	14	29	11	1	2	0	1	2	0	7	3	2b
**Patient 7**	female	58	20.8	174	17	31	4	0	1	0	0	1	0	6	2	1a
**Patient 8**	Male	64	22.3	153	14	28	8	1	0	1	1	0	1	6	3	1b
**Patient 9**	female	52	20.8	198	26	44	6	1	0	0	1	0	0	6	2	1a
**Patient 10**	Male	71	22.8	186	20	34	8	2	1	0	2	1	0	7	3	1b
**Patient 11**	Male	74	19.7	175	16	32	7	0	0	1	0	0	1	7	2	1a

## Discussion

During the surgical procedure for rectal cancer, thorough lymph node dissection is key to achieving radical surgery. Increasing the number of lymph nodes dissected and the detection rate of positive lymph nodes holds important implications for the accurate staging of a patient’s tumour, selection of subsequent treatment plans and judgement of prognosis. The lymphatic drainage of mid-low rectal cancer can simultaneously occur upwards, downwards and laterally, with the lateral pathways of metastasis following the course of the distal internal iliac artery to the perivascular area of the internal iliac vessels and the obturator internus, involving the internal iliac, obturator, external iliac and common iliac lymph nodes. Gerota et al. [1] were the first to propose in 1895 that LLNM exists in mid-low rectal cancer. Subsequent pathological and anatomical studies have further confirmed the evidence of LLNM in mid-low rectal cancer. Research reports state that the rate of LLNM in mid-low rectal cancer is approximately between 10% and 25% [2]. Conventional TME resection includes lymph nodes draining upwards and downwards but not lateral lymph nodes outside the mesorectum. Retrospective studies have shown that for patients with rectal cancer who only receive neoadjuvant therapy and TME surgery, LLNM is an important risk factor for local recurrence [3]. Amongst patients with pelvic recurrence after rectal cancer surgery, the proportion of lateral-type recurrence can be as high as 64.6%-82.7% [4]. The Japanese prospective randomised controlled study JCOG 0212 demonstrated that TME combined with LLND can considerably reduce the probability of lateral-type local recurrence [5]. Therefore, for patients clinically suspected or diagnosed with metastasis, merely undergoing neoadjuvant therapy and TME surgery is insufficient, and the combination of these treatment methods with LLND should be considered. Currently, imaging examinations remain the primary means of diagnosing preoperative LLNM. In clinical practice, MRI is considered to be the main method for assessing primary rectal tumours and lateral lymph nodes. However, MRI can also result in misdiagnosed and missed lymph nodes. According to our center’s previous experience, preoperative MRI is sometimes not a good judge when the metastatic lymph node diameter is short. Simultaneously, the complex anatomical structures of the pelvic sidewall, narrow pelvic spaces and tissue oedema caused by radiochemotherapy, amongst other factors, drastically increase the difficulty of locating and clearing rectal lateral lymph nodes under laparoscopy. In the past, when we encountered this situation, it was often difficult to locate and dissect the lymph nodes during the operation, and to identify the blood vessels and nerves. Therefore, the utilisation of lymph node localisation methods with increased precision to assist in the clearance of rectal lateral lymph nodes has become an important problem to address. Tracer imaging technologies that have emerged in recent years are gradually showing their value in solving the above issue.

ICG is a near-infrared contrast agent that emits long-wavelength near-infrared light when irradiated with exogenous light in the wavelength range of 750–800 nm. Given that ICG emits light in the near-infrared spectrum, it is generally not interfered with by the spontaneous fluorescence that may be produced by blood components, such as haemoglobin and water. On the basis of this characteristic, the ICG molecular fluorescence imaging system combines fluorescence reception imaging and fluorescence excitation to create ICG fluorescence images through a computer image processing system, highly sensitive near-infrared fluorescence camera and near-infrared excitation light source. ICG has been used for vascular imaging and functional testing since the 1950s and has gradually been applied to lymph node tracing, blood flow assessment and tumour localisation. As a result of the real-time in vivo imaging feature of ICG, surgeons can closely locate and clear tumour-draining lymph nodes under near-physiological conditions. Lymph node tracing with ICG generally involves multiple submucosal injections around the tumour under colonoscopy preoperatively or subserosal injections around the tumour intraoperatively. After injection around the tumor, the ICG will follow the lymphatic reflux and accumulate in metastatic lymph nodes. Nagata et al. [6] were the first to apply ICG to lymph node tracing in colorectal cancer in 2006, achieving good results and showcasing the considerable application prospects of ICG. Hirche et al. [7] indicated that ICG can enhance lymph node identification, facilitating the intraoperative dissection of occult, micrometastatic lymph nodes. Nishigori et al. [8] found that the use of ICG can intraoperatively display positive lymph nodes with a diameter of >5 mm, altering the scope of lymph node dissection in 23.5% of patients. Reports showed that the use of ICG can detect lateral lymph nodes in 92% of patients. Kawahara et al. [9] used ICG for sentinel lymph node tracing between the internal iliac vessels and pelvic plexus nerves, finding that in 40 patients negative for sentinel lymph nodes, none showed LLNM. This finding illustrates that ICG fluorescence imaging technology can predict LLNM during rectal cancer surgery and guide LLND. LLND alone can easily cause injury to the vessels and nerves of the pelvic sidewall, increasing the postoperative incidence of urinary dysfunction and reducing the patient’s quality of life [10]. ICG fluorescence imaging technology enhances the tissue signal background ratio, enabling the visualization of lymphatic flow by administering ICG locally. According to our research, this technique improves the identification accuracy of lymph nodes and assists surgeons in distinguishing between lymphatic vessels, lymph nodes, and non-lymphatic soft tissues. Consequently, it minimizes the occurrence of incorrect surgical procedures at inappropriate levels and reduces potential damage to blood vessels and nerves. The fundamental principle behind this approach is to prevent accidental bleeding and injury during LLND.

In this study, all 11 patients were successfully injected with ICG submucosally before surgery via colonoscopy and the imaging of lateral lymph nodes during surgery. No instance of contrast agent extravasation was observed in all patients. The intraoperative imaging effect was good, demonstrating the role and advantages of ICG fluorescence imaging technology in accurately locating lymph nodes and guiding lymph node dissection in laparoscopic surgery for rectal cancer. Furthermore, no intraoperative injuries to vessels and autonomic nerves occurred in all patients, and postoperative recovery was good, reflecting the safety and feasibility of this method. In terms of lymph node dissection, almost all patients’ lateral lymph nodes identified by ICG during surgery sent for inspection were positive, indicating that ICG can ensure the detection of lateral lymph nodes.

This study has several limitations: (1) The inclusion of only 11 patients represented a relatively small sample size, diminishing the statistical power to some extent. (2) This study only reported a single case series and lacked a comparison with conventional laparoscopic lateral lymph node dissection, making it difficult to fully highlight the advantages of ICG. (3) The relatively short follow-up period, limited to 30 days postoperatively, neglected long-term complications. The durability of the short-term advantages observed in the study for long-term benefts remains uncertain, necessitating additional follow-up in subsequent phases. Future endeavors will focus on expanding sample size, extending the follow-up duration, conducting controls, and initiating prospective single-center studies to further validate the study’s findings, paving the way for subsequent multi-center investigations.

## Conclusions

ICG fluorescence imaging technology has crucial clinical implications in the diagnosis and treatment of laparoscopic rectal cancer. It can assist in lymph node tracing and enable precise and effective surgery during LLND. Moreover, it is safe and feasible. Although it is a promising technique for performing guided lateral lymphadenectomy, it is not currently a standard of care. Future studies will further substantiate the value of ICG in the dissection of lateral lymph nodes in rectal cancer.

## Supporting information

S1 Dataset (XLS)
